# Apical ballooning syndrome: a case report

**DOI:** 10.1186/1756-0500-5-698

**Published:** 2012-12-27

**Authors:** Konstantinos M Lampropoulos, Dimitrios Kotsas, Themistoklis A Iliopoulos

**Affiliations:** 1 Department of Cardiology, Polyclinic General Hospital of Athens, 31, L. Porfyra str, Athens, 16673, Greece; 2 Department of Cardiology, 251 AirForce General Hospital of Athens, Athens, Greece

**Keywords:** Apical ballooning syndrome, Stress cardiomyopathy, Acute coronary syndrome

## Abstract

**Background:**

Apical ballooning syndrome mimics acute coronary syndromes and it is characterized by reversible left ventricular apical ballooning in the absence of angiographically significant coronary artery stenosis.

**Case presentation:**

This is a case of a 40-year-old Caucasian male without any health related problems that was submitted to an urgent coronary angiography because of acute chest pain and marked precordial T-wave inversions suggestive of acute myocardial ischemia. Coronary angiography showed no significant stenosis of the coronary arteries. Left ventriculography showed systolic apical ballooning with mild basal hypercontraction.

**Conclusion:**

Physicians should be aware of the presentation of apical ballooning syndrome, and the chest pain after following acute stress should not be readily attributed to anxiety.

## Background

Apical ballooning syndrome, also known as Takotsubo cardiomyopathy, apical ballooning cardiomyopathy, stress-induced cardiomyopathy, Gebrochenes-Herz-Syndrom, or simply stress cardiomyopathy, is a type of non-ischemic cardiomyopathy in which there is a sudden temporary weakening of the myocardium. As this weakening can be triggered by emotional stress, such as the death of a loved one, a break-up, or constant anxiety, the condition is also known as the broken heart syndrome. Stress cardiomyopathy is a well-recognized cause of acute heart failure, lethal ventricular arrhythmias, and ventricular rupture [1]. It mimics acute coronary syndromes and is characterized by reversible left ventricular apical ballooning in the absence of angiographically significant coronary artery stenosis. Studies report that 1.7-2.2% of patients originally treated as suspected acute coronary event, were subsequently diagnosed with apical ballooning syndrome. In Japanese, “tako-tsubo” means “fishing pot for trapping octopus,” and the left ventricle of a patient diagnosed with this condition indeed resembles that shape [2]. This is a presentation of a 40-year-old Caucasian male without a pertinent medical history that was referred for an urgent coronary angiography because of acute chest pain and marked precordial T-wave inversions suggestive of acute myocardial ischemia. Coronary angiography revealed no significant stenoses of the coronary arteries. Left ventriculography showed systolic apical ballooning with mild basal hypercontraction.

### Case presentation

**Figure 1:**
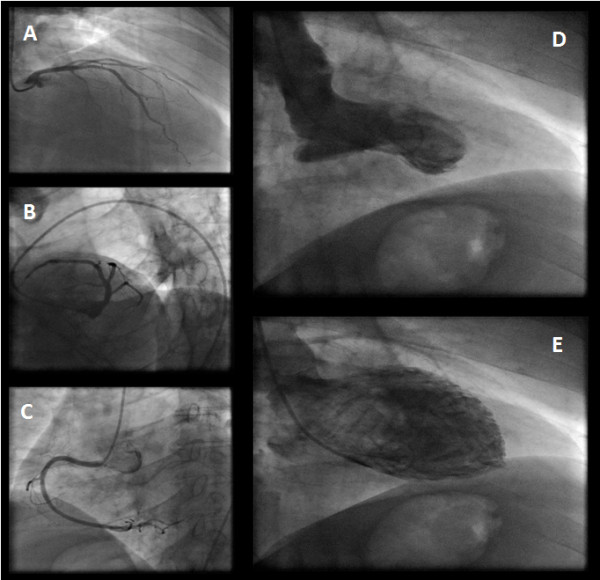
**Coronary angiography showed no significant stenosis of the coronary arteries (left coronary artery[A,B] and right coronary artery [C]).** Left ventriculography showed systolic apical ballooning with mild basal hypercontraction **[D**,**E]**.

A 40-year-old Caucasian male without a pertinent medical history was referred for an urgent coronary angiography because of acute chest pain and marked precordial T-wave inversions suggestive of acute myocardial ischemia. Cardiac catheterization was performed within an hour of admission and was negative for any significant stenosis. [Figure 1A,B,C].

Left ventriculography showed systolic apical ballooning with mild basal hypercontraction. [Figure 1D,E] Levels of creatine kinase-MB and troponin Tn(I) were mildly elevated. One month later, echocardiography showed complete resolution of the wall motion abnormality. It is of note that ten years ago, the patient experienced an episode of acute chest pain following intense emotional stress due to the unexpected death of a loved one. Regarding the presenting case, emotional stress due to occupational mishaps and subsequent unemployment had preceded the onset of symptoms.

Once the diagnosis of apical ballooning syndrome has been made, aspirin therapy was discontinued. **B**-Blocker therapy was continued, especially as an excess of catecholamines may play a significant role in precipitating the syndrome. Our patient remains well six months after his index admission.

## Discussion

The apical ballooning syndrome is an intriguing medical condition as it mimics acute coronary syndromes. Similarities in presentation can in fact mimic a classic heart attack.

Common symptoms include chest pain, shortness of breath and dyspnoea, arrhythmias or generalized fatigue. The symptoms are often preceded by an intense physical or emotional event as in our patient. Clinically, these patients tend to present similarly to the classic form. Potential triggers are news of an unexpected death of a loved one, a frightening medical diagnosis, domestic violence, financial catastrophy and severe health problems such as an asthma attack, infection, acute trauma and major surgeries. [3] Interestingly, even benign or “pleasant” exciting events can induce the syndrome. However, a case of Takotsubo cardiomyopathy was recently reported that was triggered by watching a 3D action film, the first published association between the syndrome and visual stimulation [4].

The diagnosis of apical ballooning syndrome is based on the pertinent clinical presentation and differential diagnosis should always include acute coronary syndromes. Guidelines for the diagnosis of this often puzzling condition can be found in the current medical literature [5]. The modified Mayo Clinic criteria for diagnosis of apical ballooning syndrome can be applied to a patient at the time of presentation and must contain the following 4 aspects [6]:

Transient hypokinesis, dyskinesis, or akinesis of the left ventricular midsegments, with or without apical involvement; the regional wall-motion abnormalities extend beyond a single epicardial vascular distribution, and a stressful trigger is often, but not always, present.

Absence of obstructive coronary disease or angiographic evidence of acute plaque rupture.

New electrocardiographic abnormalities (either ST-segment elevation and/or T-wave inversion) or modest elevation in cardiac troponin level.

Absence of pheochromocytoma or myocarditis.

It is noted that cardiac markers, specifically troponin I and T, are elevated in 90% of patients with apical ballooning syndrome, although to a lesser magnitude than in ST-segment elevation myocardial infarction (STEMI). The brain natriuretic peptide level is also frequently elevated.

As with any patient in whom acute coronary syndrome is suspected, an elecrocardiogram should be the initial test to be obtained after presentation to the emergency department. ST-segment elevation (67-75%) and T-wave inversion (61%) are the most common abnormalities seen on the initial ECG. Ninety-five percent of ST- elevations have been found to involve the precordial leads and to be maximal in leads V_2_ -V_3_. When compared with patients with STEMI due to a left anterior descending (LAD) coronary artery occlusion, ST-segment elevations in patients with apical ballooning syndrome were significantly less marked. Moreover, an initially normal or nonspecific ECG finding can be found in 15% of patients with apical ballooning syndrome. Diffuse T-wave inversions tend to occur in the days and weeks following presentation as the ST- segments normalize. There is no reliable way to differentiate apical ballooning syndrome from STEMI based solely on the ECG findings [7,8]. Still, new criteria are now emerging in an effort to differentiate these two separate conditions [9].

Transthoracic echocardiography provides a quick method of diagnosing wall motion abnormalities typically seen in apical ballooning syndrome, specifically hypokinesis or akinesis of the midsegment and apical segment of the left ventricle (“typical takotsubo”). Most importantly, these wall motion abnormalities extend beyond the distribution of any single coronary artery. In some cases it is possible for the hypokinesias to be restricted at the midventricular segments without involvement of the apex (“atypical takotsubo”) [10]. The left ventricular ejection fraction (LVEF) can be estimated by the echocardiogram, cardiac magnetic resonance imaging (MRI), or left ventriculography. Another diagnostic modality uniquely suited for establishing the diagnosis of apical ballooning syndrome is cardiac magnetic resonance imaging. It accurately visualises regional wall motion abnormalities, quantifies ventricular function, and identifies reversible injury to the myocardium by the presence of edema/inflammation and the absence of necrosis/fibrosis. This technology may give new insight into the pathophysiology of apical ballooning syndrome and be of potential use at acute presentations, improving recognition rates and clinical outcomes. In addition to evaluating wall-motion abnormalities and LVEF, cardiac MRI is able to differentiate apical ballooning syndrome from myocardial infarction and myocarditis based on the absence of delayed gadolinium hyperenhancement that is a hallmark of the former conditions.

Ultimately, the diagnosis of apical ballooning syndrome is confirmed at the cardiac catheterization laboratory. Normal or mildly atherosclerotic vessels are usual findings, although rarely significant obstructive coronary artery disease mat coexist by virtue of its prevalence at the population at risk. Some investigators have in fact hypothetised that apical ballooning syndrome may be a case of “aborted MI” where transient occlusions with spontaneous thrombus lysis in large left anterior descending arteries are the culprits for the characteristic wall motion abnormalities observed in the syndrome.

Patients should be treated at the emergency department as having an acute coronary event and expert Cardiology consultation should be sought promptly [11]. ACS treatments should not be stopped unless there is solid proof of an alternative diagnosis. Addressing the airway, breathing, and circulation; establishing intravenous access, providing supplemental oxygen and initiating cardiac monitoring should take precedence. Emphasis should be given to adequate hydration and attempts to alleviate the triggering stressor, emotional or physical. Tests at the emergency department should include electrocardiography, chest radiography, cardiac biomarker levels, brain natriuretic peptide, and any other laboratory studies deemed appropriate by the attending physician.

Treatment options for Takotsubo cardiomyopathy are largely empirical and supportive; however, when hemodynamics permit, beta blockers seem to be helpful. Most experts would favour the administration of standard heart failure medications, at least in the short term to counter the ensuing systolic dysfunction. Serial imaging studies may be necessary. Patients who are found to have left ventricular thrombus, which occurs in 5% of patients with apical ballooning syndrome, require anticoagulation.

Close follow-up care with a cardiologist in the weeks after diagnosis is recommended for patients with apical ballooning syndrome to ensure resolution of the cardiomyopathy, usually with serial echocardiograms. Thereafter, annual clinical follow-up is advised, because the long-term effects and natural history of apical ballooning syndrome are unknown [12].

The prognosis in apical ballooning syndrome is excellent, with nearly 95% of patients experiencing complete recovery within 4–8 weeks. The recurrence rate varies but is estimated at 3%. Estimates of mortality rates have ranged from 1–3.2% [13].

Complications occur in 20% of apical ballooning syndrome cases and include the following: Left heart failure with or without pulmonary edema, cardiogenic shock, left ventricular outflow obstruction, mitral regurgitation, ventricular arrhythmias, left ventricular mural thrombus formation or free-wall rupture.

## Conclusion

Physicians should be aware of the apical ballooning syndrome, and chest pain following a recent stressor should not readily be attributed to anxiety. It is also important to bear in mind that patients with apical ballooning syndrome do not usually have cardiac risk factors, but still their chest pain should be taken seriously. Also, patients may present to the emergency department after a natural disaster, and health care providers should be aware of apical ballooning syndrome.

## Consent

Written informed consent was obtained from the patient for publication of this manuscript and accompanying images. A copy of the written consent is available for review by the Editor-in-Chief of this journal.

## Competing interests

The authors declare that they have no competing interests.

## Authors’ contributions

KML and TAI contributed to the manuscript, performed the coronary angiography. KML and DK contributed to the manuscript, to the interpretation of the data and manuscript preparation. All authors read and approved the final manuscript.

## References

[B1] Gianni M Dentali F Grandi AM Sumner G Hiralal R Lonn E Apical ballooning syndrome or takotsubo cardiomyopathy: a systematic review Eur Heart J 2006 27 13 1523 9 10.1093/eurheartj/ehl032 16720686

[B2] Virani SS Khan AN Mendoza CE Ferreira AC de Marchena E Takotsubo cardiomyopathy, or broken-heart syndrome Tex Heart Inst J 2007 34 1 76 9 17420797 PMC1847940

[B3] Nobrega S Brito D The “Broken Heart Syndrome”: State of The Art Rev Port Cardiol 2012 31 9 589 596 22795894 10.1016/j.repc.2012.02.014

[B4] Taylor M Amin A Bush C Three-dimensional entertainment as a novel cause of takotsubo cardiomyopathy Clin Cardiol 2011 34 678 80 10.1002/clc.20950 21887691 PMC6652671

[B5] Kawai S Kitabatake A Tomoike H Guidelines for diagnosis of takotsubo (ampulla) cardiomyopathy Circ J 2007 71 6 990 2 10.1253/circj.71.990 17527002

[B6] Pilgrim TM Wyss TR Takotsubo cardiomyopathy or transient left ventricular apical ballooning syndrome: A systematic review Int J Cardiol 2008 124 3 283 92 10.1016/j.ijcard.2007.07.002 17651841

[B7] Sharkey SW Lesser JR Menon M Parpart M Maron MS Maron BJ Spectrum and significance of electrocardiographic patterns, troponin levels, and thrombolysis in myocardial infarction frame count in patients with stress (tako-tsubo) cardiomyopathy and comparison to those in patients with ST-elevation anterior wall myocardial infarction Am J Cardiol 2008 101 12 1723 8 10.1016/j.amjcard.2008.02.062 18549847

[B8] Carrillo A Fiol M Garcia Niebla J Bayes De Luna A Electrocardiographic differential diagnosis between Takotsubo syndrome and distal occlusion of LAD is not easy J Am Coll Cardiol 2010 56 19 1610 1611 author reply 1611 10.1016/j.jacc.2010.07.020 21029881

[B9] Kosuge M Ebina T Hibi K Morita S Okuda J Iwahashi N Simple and accurate electrocardiographic criteria to differentiate takotsubo cardiomyopathy from anterior acute myocardial infarction J Am Coll Cardiol 2010 55 22 2514 6 10.1016/j.jacc.2009.12.059 20510222

[B10] Kurowski V Kaiser A von Hof K Killermann DP Mayer B Hartmann F Schunkert H Radke PW Apical and midentricular transient left entricular dysfunction syndrome (tako-tsubo cardiomyopathy): frequency, mechanisms and prognosis Chest 2007 132 809 10.1378/chest.07-0608 17573507

[B11] Bybee KA Kara T Prasad A Lerman A Barsness GW Wright RS Rihal CS Systematic review: transient left ventricular apical ballooning: a syndrome that mimics ST-segment elevation myocardial infarction Ann Intern Med 2004 141 858 65 15583228 10.7326/0003-4819-141-11-200412070-00010

[B12] Dorfman TA Iskandrian AE Takotsubo cardiomyopathy: State-of-the-art review J Nucl Cardiol 2009 16 1 122 34 10.1007/s12350-008-9015-3 19152137

[B13] Donohu D Movahed MR Clinical characteristics, demographics and prognof transient left ventricular apical ballooning syndrome Heart Fail Rev 2005 10 4 311 6 10.1007/s10741-005-8555-8 16583180

